# Early-Life Exposure to Commercial Formulation Containing Deltamethrin and Cypermethrin Insecticides Impacts Redox System and Induces Unexpected Regional Effects in Rat Offspring Brain

**DOI:** 10.3390/antiox12051047

**Published:** 2023-05-05

**Authors:** Fatiha Mekircha, Donatella Fedeli, Cinzia Nasuti, Hadjer Kecies, Rosita Gabbianelli, Laura Bordoni

**Affiliations:** 1 Laboratory of Biotechnology, Environment and Health, Faculty of Natural and Life Sciences, University Mohammed Seddik Ben Yahia, Jijel 18000, Algeria; mekirchafatiha@yahoo.fr; 2 Unit of Molecular Biology and Nutrigenomics, School of Pharmacy, University of Camerino, 62032 Camerino, MC, Italy; donatella.fedeli@unicam.it (D.F.); laura.bordoni@unicam.it (L.B.); 3 Unit of Pharmacology, School of Pharmacy, University of Camerino, 62032 Camerino, MC, Italy; cinzia.nasuti@unicam.it; 4 Laboratory of Natural Science and Materials (LSNM), Institute of Science and Technology, Abdelhafid Boussouf, University Center Mila, Mila 43000, Algeria; h.kecies@centre-univ-mila.dz

**Keywords:** deltamethrin, cypermethrin, oxidative stress, α-synuclein, antioxidant enzymes, rats

## Abstract

Several studies have shown that the oxidative impact of pesticides is most prevalent in rural environments where they are intensively used. At different levels, pyrethroids are reported to promote neurodegeneration; they share the ability to promote oxidative stress, and to induce mitochondrial impairments, α-synuclein overexpression and neuronal cell loss. The present study evaluates the impact of early-life exposure to a commercial formulation containing deltamethrin (DM) and cypermethrin (CYP) at a dose of 1/100 LD50 (1.28 and 2.5 mg/kg, respectively). Rats aged 30 days old, treated from the 6th to the 21st day of life, were tested for brain antioxidant activity and α-synuclein levels. Four regions of the brain were analyzed: the striatum, cerebellum, cortex and hippocampus. Our data demonstrated a significant increase in catalase (CAT), superoxide dismutase (SOD) and glutathione (GSH) antioxidant levels in the brain regions compared to the controls. Pups exhibited no significant changes in protein carbonyl levels and lipid peroxidation. Striatal α-synuclein expression was significantly reduced in the rats exposed to DM + CYP, while the treatment resulted in a non-significant increase in the other brain areas. These findings indicate unexpected effects of postnatal treatment with the commercial formulation containing DM and CYP on brain redox state and α-synuclein expression, suggesting an adaptive response.

## 1. Introduction

Due to their sufficient photostability, low environmental persistence, or both of these qualities, insecticides of the pyrethroid class are frequently utilized in agriculture and domestic pest control [1]. Pyrethroids have effectively replaced organophosphates and methylcarbamate insecticides in crop protection. They are synthetic compound analogues of pyrethrins, which are natural insecticides present in the flower of the genus Chrysanthemum cinerariifolium. The inclusion of an α-cyano group on the 3-phenoxybenzyl alcohol moiety, as in deltamethrin (DM) and cypermethrin (CYP), produced type II class pyrethroids, that have a particularly strong insecticidal potency against a broad spectrum of insects and an appreciable safety margin [1,2].

However, both epidemiological and laboratory studies have summarized the toxic effects of α-cyano derivatives [3,4,5]. Many of the commonly used pyrethroids have significant cardiotoxic, immunotoxic, reproductive and neuronal effects on mammals [6,7,8,9,10]. Epidemiological evidence suggests that exposure to pyrethroids is a risk factor for the impairment of the redox system and the development of neurodegeneration [11,12,13,14,15,16]. Moreover, it can cause the abnormal growth of fibrillar α-synuclein protein in Lewy bodies throughout various brain regions [17]. At different doses, pyrethroid brain toxicity is associated with the promotion of oxidative stress. For example, exposure to deltamethrin and other pyrethroids induces free radical generation, leading to neurodegenerative disorders and neurodevelopmental deficits [18,19]. Furthermore, cypermethrin can cross the blood–brain barrier and cause mitochondrial dysfunction and oxidative damage, as well as α-synuclein aggregation [20].

Concerns about the link between pesticide exposure and neuronal damage are growing [11,21,22,23]. The developing nervous system’s vulnerability to environmental chemicals is primarily challenged by the duration of exposure, and is accelerated if there is exposure during the organ’s development [6,7,17]. In rats, neural development begins from the second week of gestation and proceeds until postnatal day 21; thus, this period represents a window of susceptibility for harmful environmental exposure [17].

Evidence from the literature shows that early-life permethrin exposure in rats induces long-term effects on the hippocampus, such as the impairment of long-term memory storage and of the development of synapses [24]. Furthermore, permethrin administration to rat offspring during early life, from the 6th to the 21st day, induces alterations in the striatum [25], heart and plasma [26]. Carloni et al. demonstrated that early-life permethrin treatment at a dose close to the NOAEL (25 mg/kg) reduces both NO and calcium levels, as well as Nurr1 gene and protein expression, in the striata of rats [27]. Recent studies on the same animal model have shown that early-life exposure to permethrin induces epigenetic changes associated with lower dopamine levels in the striatum that can be inherited by the progeny [28,29].

On the contrary, it has been described that low doses of pollutants can induce adaptive processes in various models (i.e., cell line, mammals, *Caenorhabditis elegans*, *Drosophila* melanogaster) [30,31,32]. Such an adaptation is also called hormesis [30]. All life forms appear to be able to exhibit adaptive stress responses through a mechanism of direct enzyme activation/deactivation. These adapted cells are thus significantly more resistant to oxidative stress [33,34]. A hormetic-like effect of oral acute treatments with different low doses of the neonicotinoid clothianidin on behavioral responses to the sex pheromone in the moth *Agrotis ipsilon* has been addressed by Rabhi et al. [33], with the unexpected effect of improving orientation. Additionally, as pointed out in the study of Pickering et al. [34] cultured mammalian cells adapt to chronic and/or repeated stress by upregulating the antioxidant protective systems.

The toxicity of individual pyrethroids (deltamethrin, cypermethrin, bifenthrin, bioallethrin and others) at different life stages has been thoroughly examined and evaluated mechanistically [5,9,13]. The complication lies in the assessment of the response to combined exposures to pyrethroids of different classes.

Thus, this work aims to investigate whether early-life exposure to a commercial formulation containing a mixture of DM and CYP, selected because of their extensive use, can influence the brain’s antioxidant capacity or induce a different response that prevent oxidative stress and alterations to neurodegenerative markers, such as α-synuclein, in the cerebral areas of rat offspring.

## 2. Materials and Methods

### 2.1. Chemicals

Deltamethrin in the commercial formulation Decis 25EC, composed of 2.8% technical grade deltametrin in emulsifiable concentrate and purchased from Bayer Crop Sciences (Spain), was used in this study. Additionally, we utilized a commercial formulation of cypermethrin, marketed as “Sherpa 25 EC”, containing 250 g/L technical grade cypermethrin in emulsifiable concentrate and obtained from CAPISCOL (Agro Consulting International, Algiers, Algeria). All the other chemicals and reagents were of analytical grade and purchased from Sigma chemicals (Aldrich Chemical Company, St. Louis, MO, USA).

### 2.2. Animals

**Figure 1:**
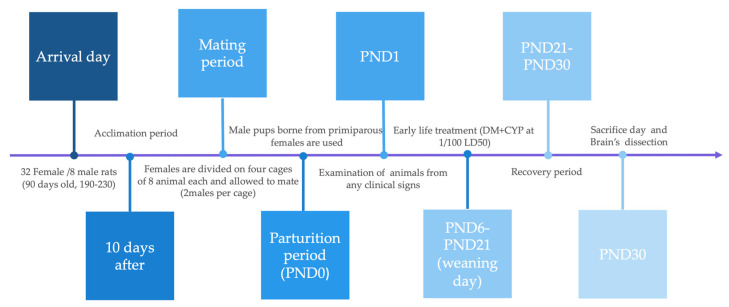
Timeline of all experimental procedures.

All experimental procedures were performed in accordance with the Guide for the Care and Use of Laboratory Animals established by the European Communities Council (86/609/ECC). The study was approved by the ethical committee of the Algerian Association of Experimental Animal Sciences (88–08/1988) (AASEA/USTHB, February 2015). Forty healthy adult male and female Albino Wistar rats aged about 90 days (190–230 g) were obtained from the Pasteur institute (Algiers, Algeria). After 10 days of acclimation, thirty-two (n = 32) female rats were allowed to mate with eight (n = 8) adult male rats. Animals were subjected to a 12 h light/dark cycle, maintained at a room temperature of 18–22 °C and relative humidity of 40–50%, and fed a standard laboratory diet and water ad libitum. In the study, we used rat pups born from primiparous females. The parturition day was considered postnatal day zero (PND0). On PND1, all litters were examined externally for the presence of gross abnormalities, and were sexed and weighed; female pups were excluded from this study and addressed in another study (Ethical Committee of the Algerian Association of Experimental Animal Sciences (88–08/1988), (AASEA/USTHB, February 2015), while male pups were each assigned to a dam until weaning (PND21). At 6 days of age, litters (n = 12) were randomly assigned to two groups identified as the control group (n = 6 offspring rats) and the treated group (n = 6 offspring rats) (Figure 1), and treated as reported in Section 2.3.

### 2.3. Early-Life Treatment and Sample Collection

Twelve (n = 12) male offspring rats were used in the present study: 6 controls (corn oil) and 6 treated with the commercial formulation containing DM + CYP (diluted in corn oil). Corn oil was used to dilute the formulation to achieve the considered test dose level of DM and CYP. Pesticides in the commercial formulations were in emulsifiable concentrated organic–aqueous solution containing 1% tetrapropylene benzene sulfonic acid, 1% methylpropanol and 25% light aromatic solvent naphtha (petroleum). In this study, we did not include a solvent group since previous works have not shown significant differences between the control group and the solvent group [5,35]. Commercial formulations containing pyrethroids were dissolved in corn oil and administered orally (4 mL/kg) at a dose of 2.5 mg/kg body weight of cypermethrin and 1.28 mg/kg body weight of deltamethrin (1/100 LD50). Drugs were administered orally using a flexible plastic feeding tube securely attached to a syringe (25–30 mm, 3–6 µL) purchased from INSTECH, USA. The feeding tube was appropriately chosen, taking in account body weight, drugs and esophageal length to prevent perforation. Safer medical-grade propylene tube ends with a soft rounded bulbous tip were used in this study. The doses of DM and CYP used in this work were selected based on previous studies [35,36,37,38]. In particular, the selected dose or CYP (2.5 mg/kg body weigh) was used previously during early life, and other targets were studied [38]. The tested concentrations were obtained by calculating the percentage of the active ingredient (DM and CYP) in the commercial formulation. Different concentrations of test formulation were freshly prepared daily and administered from PND6 to PND21, once a day, in the morning. Rats in the control group received only corn oil in an identical manner. The volume of the compounds administered was adjusted daily on the basis of body weight, which was measured during the dosing period. Throughout the treatment period, all animals were checked daily for any clinical signs or mortality, and we did not observe any abnormal changes in different experimental animals. After treatment, the animals were allowed to recover for one week. At 30 days old, all male pups were sacrificed under CO_2_ asphyxia upon exposure to CO_2_ without removing the animals from their home cage. It is a rapid and ethical method because the animals are not stressed due to handling or being moved to a new environment. The brain tissue was carefully removed, and the striatum (ST), cerebellum (CB), cortex (CR) and hippocampus (HP) were rapidly hand-dissected at 4 °C according to the method of Nasuti et al. [39]. The brain was carefully removed from the skull, taking off the meninges, and it was then placed ventrally on the convex cover of a Petri dish on ice. Individual brain structures (CB, CR, HP and ST) were dissected using reference coordinates obtained from the rat brain atlas [40]. Firstly, the CB was dissected away from the brainstem with incisions through the cerebellar peduncles. Secondly, the CR overlying the dorsal hippocampi was removed with a dissecting needle and spatula, starting from the interhemispheric fissure. The CR was removed after partial disruption of the corpus callosum. Next, a cut was made with the tip of a scalpel along the posterior and anterior edges of the HP in order to separate them from white matter, and with spatula, they were picked up. Finally, the brain, with the ventral side facing the glass dish, was dissected in coronal slices according to brain atlas coordinates, and both the right and left ST were picked up. Tissues were immediately frozen at −80 °C until their use in the oxidative stress study and Western blot analysis to identify the protein expression of alpha-synuclein.

### 2.4. Sample Preparation

The four brain regions analyzed in the present study were the striatum (ST), cerebellum (CB), cortex (CR) and hippocampus (HP). Following sample collection, a portion of each tissue was individually homogenized in 50 mM cold phosphate-buffered saline at pH 7.4 and at 4 °C. Homogenates were normalized using the method of Lowry et al. [41]. The second part of each brain region was individually pooled, and tissues were processed to study the protein expression of α-synuclein.

### 2.5. Oxidative Stress Study in the Brain

#### 2.5.1. Superoxide Dismutase Determination

An SOD assay was performed on the extracts obtained, and we treated the normalized brain region homogenates with chloroform–ethanol (1:1) solution, as reported by Concetti et al. in 1976 [42], in the two groups: the control (n = 6) and treated rats (n = 6). The SOD activity was assessed on an ethanol fraction using the adrenaline method developed by Misra and Fridovich in 1972 [43]. The reaction mixture contained 0.05 M carbonate buffer, 0.1 M EDTA, pH 10.2, 0.05 M adrenaline and 50 µL of ethanol extract of samples normalized to 1 mg/mL protein. The enzyme activity was measured spectrophotometrically at 480 nm and at 37 °C following the inhibition of adrenaline’s reaction to adrenochrome by SOD. One unit of SOD was defined as the quantity of enzyme necessary to induce 50% inhibition of the adrenaline–adrenochrome reaction. The results were calculated using a standard curve in the brain regions analyzed, and they are reported as μg of SOD in 1 mg of protein.

#### 2.5.2. Catalase Activity Determination

CAT activity was determined using Luck’s method developed in 1974 [44] in the control (n = 6) and treated rats (n = 6). This assay was performed using 3 mL 0.066 M potassium phosphate buffer at pH 7.0, 40 µL of 9 M H2O2, and the ST, CR, HP and CB homogenates of control and treated pups (n = 6 in each group), normalized to 1 mg/mL protein. The change in absorbance was measured spectrophotometrically at 240 nm at 20 °C. Enzyme activity was assessed using a molar extinction coefficient for H2O2 of 81 M^−1^ cm^−1^. The results were calculated using a standard curve in the brain regions analyzed, and they are reported as μg of CAT in 1 mg of protein.

#### 2.5.3. Glutathione Determination

GSH content in the various brain regions’ homogenates obtained from the control (n = 6) and treated rats (n = 6) was calculated using the method previously described by Butler et al. [45]. Samples were normalized to a concentration of 0.8 mg/mL protein using the method of Lowry et al. [42]. The sulfhydryl reagent 5,5′-dithio-bis (2-nitrobenzoic acid) (DTNB) was used to measure oxidized GSH using a spectrophotometer at a wavelength of 412 nm with respect to its standard.

#### 2.5.4. Lipid Peroxidation Determination

Lipid peroxides were detected in the control (n = 6) and treated rats (n = 6), in different brain regions, using the fluorescent probe DPPP, which localizes in the membrane and becomes highly fluorescent when oxidized by hydroperoxides, as described by Takahashi et al. [46]. A total of 0.4 mg of protein taken from every sample of the treated and control brain homogenates (n = 6 in each group) was adjusted to 1 mL with polyphosphate buffer (PBS; pH 7.4). Subsequently, 1 µL of 1 µM DPPP was added, and samples were incubated at 37° for 5 min in the dark. The fluorescence intensities of the samples were detected with a Hitachi 4500 spectrofluorometer using 351 and 380 nm as excitation and emission wavelengths, respectively.

#### 2.5.5. Protein Carbonyl Determination

PC content was measured in the cortex, cerebellum, hippocampus and striatum samples from the control (n = 6) and treated rats (n = 6), as previously reported by Soglia et al. [47] based on the reactivity of carbonyl groups with 2,4-DNPH. A total of 0.5 mL of 10 mM DNPH dissolved in 2.5 M HCl was added to each sample, which contained a 0.5 mg/mL protein concentration. Samples were incubated at room temperature for 1 h with continuous shaking; blank reactions lacked only DNPH. A total of 0.5 mL of 20% TCA was added in order to precipitate the proteins, and then, the samples were centrifuged at 4000 rpm for 5 min. The pellet containing protein was washed three times with ethanol:ethyl acetate (1:1) and finally dissolved in 0.6 mL of 6 M guanidine HCl (pH 7.4). Carbonyl concentration, expressed as nmoles/mg protein, was determined spectrophotometrically at a wavelength of 370 nm, and calculated using a molar absorption coefficient of 22,000 mmol^−1^ cm^−1^.

### 2.6. Immunoblotting Analysis

Western blot analysis was carried out in various brain regions tissues in the control (n = 6) and treated rats (n = 6), to analyze effects of DM and CY on α-synuclein expression. To prepare the samples, pools of ST, CB, CR and HP obtained from the control and treated rats (n = 6 in each group) were lysed using RIPA buffer (1% NP40, 0.5% Na-deoxycolic acid and 0.1% SDS in phosphate-buffered saline (PBS)) containing fresh protease inhibitors. The protein concentration of lysates was quantified using the Lowry method (1951), and equal amounts (40 μg) of protein from each lysate were separated using SDS-PAGE (12%), as described by Carloni et al. [25]. The gel was electrophoretically blotted on a nitrocellulose support (Hybond C, Amersham Bioscience, Little Chalfont, UK). The nitrocellulose membrane was incubated with PBS containing 0.05% Tween 20 (PBST) and non-fat milk (5%) for 1 h at room temperature, and incubated with primary rabbit polyclonal α-synuclein antibody (Santa Cruz Inc., Dallas, TX, USA) diluted in a ratio of 1:500. After 1 h of incubation, the membrane was rinsed with PBST buffer, followed by 1 h of incubation with secondary anti-rabbit antibody diluted in a ratio of 1:5000 (KPL, Ocracoke, NC, USA). After three washes with PBST buffer, the immunoreactive bands were visualized using an ECL chemiluminescence kit. β-actin was utilized as a control for equal protein loading. Membranes were stripped and reprobed for β-actin with an anti-rabbit monoclonal antibody (Abcam plc, Cambridge, UK) diluted in a ratio of 1:3000 in PBS containing 0.05% Tween 20 and bovine serum albumin (2%). Every gel was loaded with molecular weight markers including proteins with MW from 250 to 4 kDa (Invitrogen, Waltham, MA, USA). Image capturing was performed using the KODAK Image Station 2000r System, and the band intensities of the target protein were analyzed using the ImageJ program 1.50f.

### 2.7. Statistical Analysis

All experiments were conducted in six replicates and are reported as the mean ± SD. The data were first tested for normality and group homogeneity using a QQ plot and Levene’s test. In addition, means were separated using a Student’s independent *t*-test. Differences were considered significant at a level of *p* < 0.05 using the STATISTICA 10 program.

## 3. Results

### 3.1. Antioxidant Enzymes

#### 3.1.1. Catalase Analysis

**Figure 2:**
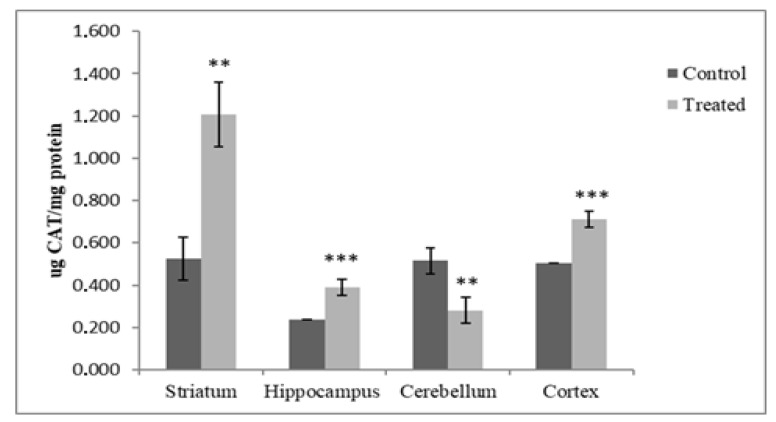
DM + CYP-induced changes in catalase concentration in all four brain regions (ST, HP, CB and CR). Data are expressed as means ± SD (n = 6). ** *p* < 0.01 and *** *p* < 0.001 when compared to control.

The evaluation of catalase concentration in the selected brain regions of offspring exposed postnatally to DM and CYP showed a significant decrease (45.43%, *p* < 0.01) in the CB. On the contrary, a significant increase of 40.71% was measured in the CR (*p* < 0.001); in the HP, there was an increase of 64.13% (*p* < 0.001); and in the ST. there was an increase of 130.34% (*p* < 0.01) compared with controls (Figure 2).

#### 3.1.2. SOD Analysis

**Figure 3:**
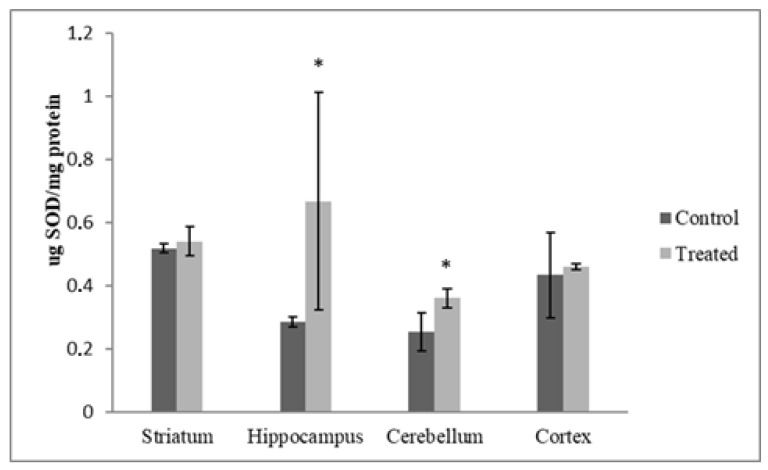
DM + CYP-induced changes in SOD concentration in HP and CB. Data are expressed as means ± SD (n = 6). * *p* < 0.05 when compared to control.

The administration of the toxicants under study caused a significant increase in the concentration of SOD in the HP (133.24%, *p* < 0.05) and CB (42%, *p* < 0.05) compared to the controls. No significant variation was observed in SOD concentration in the CR and ST (5.34%, *p* > 0.05 and 4.23%, *p* > 0.05, respectively) in comparison to the controls (Figure 3).

### 3.2. GSH Content

**Figure 4:**
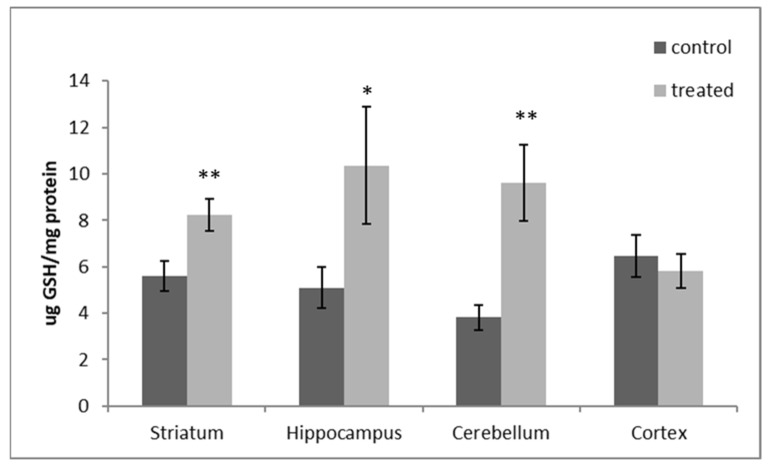
DM + CYP-induced changes in GSH levels in all four brain regions (ST, HP, CB and CR). Data are expressed as means ± SD (n = 6). * *p* < 0.05 and ** *p* < 0.01 when compared to control.

The exposure of pups to DM + CYP during the postnatal period caused a significant increase in the level of GSH in the HP (103.23%, *p* < 0.05), CB (151.78%, *p* < 0.01) and ST (46.59%, *p* < 0.01) when compared to the corresponding areas of the control rats. In the cortex, the tested pyrethroid seems to have had no effect on GSH levels in the treated group compared to the controls (10.35%, *p* > 0.05) (Figure 4).

### 3.3. Lipid Peroxidation

**Figure 5:**
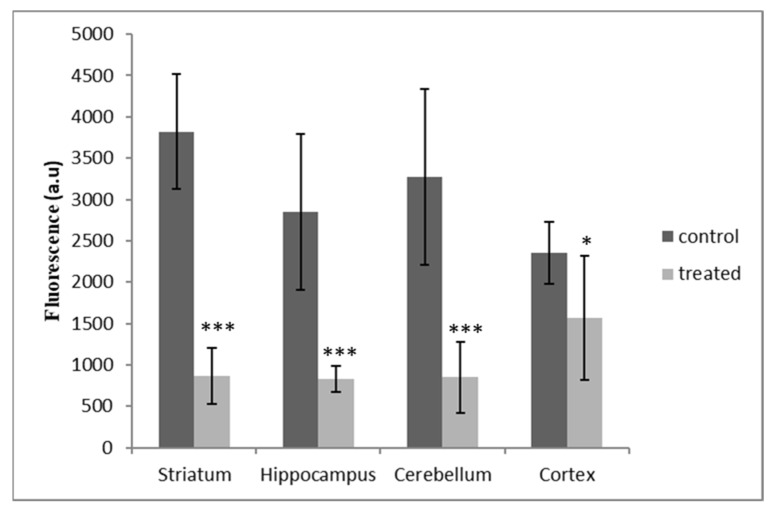
DM + CYP-induced changes in lipid peroxidation measured via DPPP fluorescence in all four brain regions (ST, HP, CB and CR). Data are expressed as means ± SD (n = 6). * *p* < 0.05 and *** *p* < 0.001 when compared to control. a.u: arbitrary unit.

Pyrethroid exposure during the postnatal period caused a significant decrease of 33.31% in DPPP fluorescence in the CR (*p* < 0.05); however, this decrease was greater and was almost equal for the rest of the regions in the treated rats when compared to the corresponding controls (70.25% (*p* < 0.001), 74.07% (*p* < 0.001) and 77.25% (*p* < 0.001) in the HP, CB and ST, respectively) (Figure 5).

### 3.4. Protein Carbonyl

**Figure 6:**
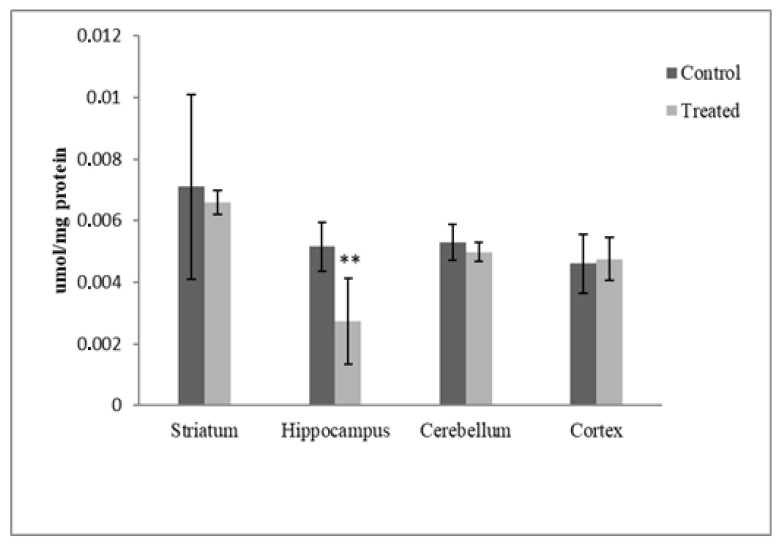
DM + CYP-induced changes in protein carbonyl levels in HP. Data are expressed as means ± SD (n = 6). ** *p* < 0.01 when compared to control.

Figure 6 shows the protein carbonyl levels in the ST, CR, HP and CB tissue homogenates obtained from the control and treated rats. A significant protein carbonyl level decrease was reported in the HPs of treated rats with respect to the control (47.08%, *p* < 0.01), while no changes were observed in their CBs, STs or CRs (*p* < 0.05).

### 3.5. Western Blot Analysis

**Figure 7:**
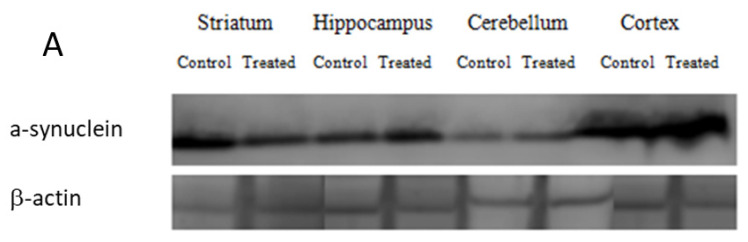

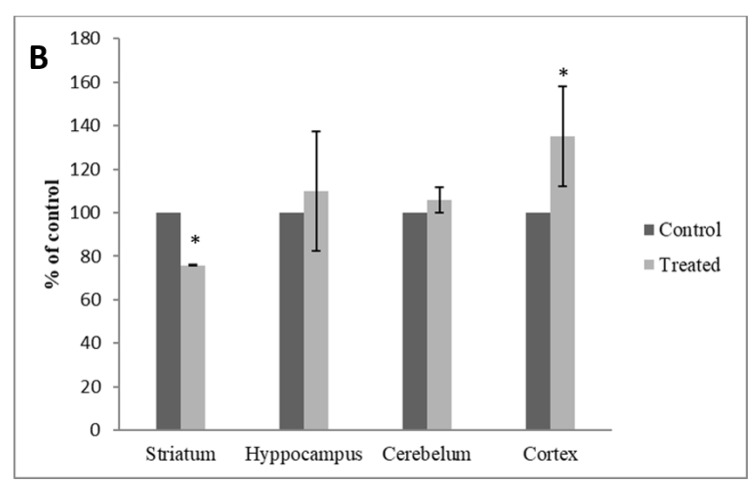
Western blot detection (**A**) and percentage of control (**B**) in the expression of α-synuclein in STs, HPs, CBs and CRs of control and DM + CYP-treated rats during postnatal period. Data are expressed as means ± SD (n = 6). * *p* < 0.05 when compared to control.

The expression of α-synuclein was assessed in the brain regions. Figure 7 shows the Western blot analysis results for the ST, HP, CB and CR pools obtained from the tissues of the control and treated rats with anti-α-synuclein antibodies. The western blot analysis revealed a significant decrease in the immunoreactivity of alpha synuclein in the ST (75.82%, *p* <0.05) compared to the control rats (100%). Similarly, the expression level of α-synuclein protein was increased in the HP (110.02%) and CB (105.88%). However, this increase was found to be statistically significant only in the CR (135.21%, *p* < 0.05) of the offspring exposed postnatally to DM + CYP.

## 4. Discussion

To counterbalance free radicals, cells possess a line of antioxidant compounds and enzymes which contribute to keeping the levels of reactive oxidants low [48,49]. Through the dismutation of superoxide anions by SOD and the conversion of hydrogen peroxide to water by CAT activity, oxidative stress can be counterbalanced [50]. In this study, in vivo exposure to deltamethrin and cypermethrin significantly increased the activity of CAT in the cortices, hippocampi, and striata of treated rats. The activity of SOD was measured in the HPs and CBs of treated rats in response to increased levels of reactive oxygen species (ROS). This is in accordance with the adaptive responses of animals to a range of stressors described in previous studies, demonstrating that antioxidant enzyme systems are upregulated and adaptable [51]. Specific responses to environmental stimuli (i.e., chemicals, radiation, food, etc.) can be observed in various cell types; for instance, mice under low X-rays showed an increase in SOD and CAT activity in their livers [52]. Additionally, in rats exposed to hydrogen peroxide, CAT, GPX and SOD activities were elevated in the tracheobronchial epithelia [51]. This phenomenon was similar to the conclusion of Pamok et al. [52], who described that heat-stressed broilers may adapt to elevated environmental temperatures and oxidative stress by enhancing their antioxidative response. In the case of low and short stressful events [51,52], organisms respond by upregulating the genes involved in the redox signaling pathways to counterbalance stressor damage and to develop resilience. Furthermore, the unexpected effects of some other components of commercial formulations can be excluded since these products have already been tested [5,37], and no effects on enzymes involved in the control of the redox system, or on targets of oxidation (i.e., lipid oxidation, GSH level), have previously been reported.

Tripeptide GSH (l-glutamyl-l-cysteinyl glycine) is the most abundant cellular antioxidant [53]. GSH is created in two steps: GSH synthase adds glycine after gamma-glutamyl-cysteine synthetase (gamma-GCS) first generates a peptide bond between glutamic acid and cysteine [54]. Through either direct interaction with reactive species or indirect interactions with glutathione transferases, GSH has a crucial role in the redox system, and it counterbalances the oxidation of protein thiol groups [55,56,57]. In the current study, the binary mixture of DM and CYP was shown to induce a significant increase in the levels of GSH (Figure 4). This can be explained considering that pups have an active antioxidative defense system to counteract ROS, as demonstrated by the increased GSH due to exposure to chemicals [56,57].

Additionally, exposure to the commercial formulation containing DM and CYP during the postnatal period caused a significant decrease in DPPP fluorescence in all the brain regions of the treated rats (Figure 5). This outcome was in accordance with the increased antioxidant activity of CAT, SOD and GSH in the treated groups; the ability of rat pups to cope with stress represents a strategy to mitigate the negative effects of low pesticide exposure.

The present study also shows that postnatal exposure to DM and CYP, accompanied by elevated levels of GSH and antioxidant enzyme activities, decreased protein carbonyl levels in the hippocampi of exposed rats, confirming its impact on redox homeostasis. On the other hand, no changes were measured between the control and treated groups in the ST, CB or CR. According to several authors, protein carbonyls are formed via the direct oxidation of amino acids (lysine, arginine, proline and threonine) [58,59]. The formation of a protein carbonyl occurs due to protein oxidation, which can be induced by environmental factors such as pesticides and can drastically alter the tertiary structure of a protein; this can result in its partial unfolding, which is associated with a rise in protein hydrophobicity that results in deleterious protein–protein interactions [60]. In this field, the regulatory role of the proteasome has been thoroughly discussed [61,62]. Proteasome inhibition occurs in a variety of CNS disorders, and neurotoxicity plays a crucial role as a mediator of oxidative stress and protein damage [61,62]. However, in the context of oxidative stress adaptation or hormesis, the retention of the proteasome’s proteolytic function is a fundamental tool by which oxidized proteins are destroyed and cells are able to resist oxidative damage [61]. In particular, the increase in20S proteasome synthesis has been pointed out as crucial in the adaptive response to oxidative stress [63].

Several studies suggest that α-synuclein tends to aggregate and accumulate in the mitochondria of human neuroblastoma cells [64,65]. Moreover, this accumulation is involved in inducing oxidative stress and decreasing mitochondrial function [66]. However, the expression of α-synuclein in specific areas can vary significantly from cell to cell [67]; dopaminergic neurons, a heterogeneous type of cell located in various brain regions, are characterized by the same neurotransmitters that express different markers depending on their location [29,66]. Consequently, discrepancies in α-synuclein expression due to differences in the treatments and parameters analyzed have been reported [68]. Upon examining the immunoblots of brain cells lysates (Figure 6), it appears that early exposure to the commercial formulation containing DM and CYP results not in significant change in α-synuclein protein level in the hippocampus and cerebellum, but in a significant increase in the cortical tissue. Instead, a significant decrease in striatal synuclein expression was measured in the present study; this result may be partially explained by the fact that it had the highest CAT value compared to the other brain regions (Figure 2). Contrasting findings are described in previous studies, showing distinct expression patterns of α-synuclein in the brain. Although the mechanisms are far from being elucidated, data show that permethrin administered to C57BL/6 mice at a dose of 200 mg/kg induced an elevation in α-synuclein expression in the striatum, about 20% above the control [69]. Additionally, a similar increase in α-synuclein immunoreactivity was observed in animals exposed to paraquat not only in the striatum, but also in hippocampus, provoking the formation of protein deposits [70,71]. Interestingly, authors have proposed that α-synuclein itself may have properties that counteract toxic injury, and its expression could affect specific stress signaling pathways linked to neuronal survival. Hashimoto et al. [72] reported that α-synuclein expression can induce resistance to in vitro hydrogen peroxide toxicity via the inactivation of c-Jun N-terminal kinase, a member of the mitogen-activated protein kinase family. This indicated that α-synuclein overexpression provides cryoprotection against paraquat-induced neurodegeneration [72].

On the other hand, a higher concentration of α-synuclein protein in different brain cell types, and the downregulation of α-synuclein in the brain regions, have been reported in some synucleinopathies; this might protect cells against oxidative stress [73] upon activating nuclear factor erythroide-2-related factor (Nrf2). As a result, it shortens α-synuclein’s half-life and speeds up clearance, resulting in reduced levels of the protein in tissues.

In conclusion, our study describes an adaptive response of brain tissue through the attenuation of oxidative stress damage, and the boosting of cellular resistance to oxidation at different levels. Significant enhancements in CAT and SOD activity and GSH levels in the brain regions were observed in offspring treated early with the commercial formulation containing DM + CYP compared to the control animals. The increase in antioxidant cell capacity could explain the decreased level of lipid peroxidation measured via low-DPPP fluorescence. In addition, we did not observe significant changes in protein carbonyl levels in pups exposed to the commercial formulation containing the pyrethroid mixture. Our results highlight decreased expression of α-synuclein in the striatum and increased expression in the cortex following treatment with the commercial formulation containing DM + CYP, while no effect on the hippocampus or cerebellum was detected. Finally, the involvement of other components in the commercial formulations should be marginal since, as reported in previous investigations [5,37], they do not impact oxidative biomarkers.

The current investigation raises several questions on the impact of postnatal exposure to pyrethroids in rats’ brains. Further molecular and cellular studies will focus on better characterizing the mechanisms underpinning the neurodegenerative alterations associated with exposure to formulations containing combined pyrethroids. Upcoming studies will be designed to evaluate the impact of the current findings on the dopaminergic nervous system; in particular, the nigrostriatal dopamine system will be studied for specific biomarkers (i.e., dopamine transporter, tyrosine hydroxylase, and vesicular monoamine transporter 2) in order to understand how the commercial formulation containing CYP and DM can perturb the dopamine system in rats, and to understand the observed perturbation of α-synuclein expression.

## Data Availability

Not applicable.

## Abbreviations

DM—commercial formulation containing deltamethrin; CYP—commercial formulation containing cypermethrin; LD_50_—Lethal dose 50; NOAEL—no observed adverse effect level; MW—molecular weight; GSH—glutathione; CAT—catalase; SOD—superoxide dismutase; EC—emulsifiable concentrate; PND—postnatal day; HP—hippocampus; CB—cerebellum; CR—cortex; ST—striatum; ROS—reactive oxygen species; CNS—central nervous system.

## References

[1] Casida J.E. (1980). Pyrethrum flowers and pyrethroid insecticides. Environ. Health Perspect..

[2] Soderlund D.M. (2012). Molecular mechanisms of pyrethroid insecticide neurotoxicity: Recent advances. Arch. Toxicol..

[3] Ismail A.A., Wang K., Olson J.R., Bonner M.R., Hendy O., Abdel Rasoul G., Rohlman D.S. (2017). The impact of repeated organophosphorus pesticide exposure on biomarkers and neurobehavioral outcomes among adolescent pesticide applicators. J. Toxicol. Environ. Health..

[4] Hocine L., Merzouk H., Merzouk S.A., Ghorzi H., Youbi M., Narce M. (2016). The effects of alpha-cypermethrin exposure on biochemical and redox parameters in pregnant rats and their newborns. Pestic. Biochem. Physiol..

[5] Mekircha F., Chebab S., Gabbianelli R., Leghouchi E. (2018). The possible ameliorative effect of *Olea europaea* L. oil against deltamethrin-induced oxidative stress and alterations of serum concentrations of thyroid and reproductive hormones in adult female rats. Ecotoxicol. Environ. Saf..

[6] Nasuti C., Carloni M., Fedeli D., Gabbianelli R., Di Stefano A., Serafina C.L., Silva I., Domingues V., Ciccocioppo R. (2013). Effects of early life permethrin exposure on spatial working memory and on monoamine levels in different brain areas of pre-senescent rats. Toxicology.

[7] Vadhana M.S.D., Arumugam S.S., Carloni M., Nasuti C., Gabbianelli R. (2013). Early life permethrin treatment leads to long-term cardiotoxicity. Chemosphere.

[8] Kumar A., Sasmal D., Sharma N. (2015). An insight into deltamethrin induced apoptotic calcium, p53 and oxidative stress signalling pathways. Toxicol. Environ. Health Sci..

[9] Syed F., Chandravanshi L.P., Khanna V.K., Soni I. (2016). Beta-cyfluthrin induced neurobehavioral impairments in adult rats. Chem. Biol. Interact..

[10] Fedeli D., Montani M., Bordoni L., Galeazzi R., Nasuti C., Correia-Sa L., Domingues V.F., Jayant M., Brahmachari V., Massaccesi L. (2017). In vivo and in silico studies to identify mechanisms associated with Nurr1 modulation following early life exposure to permethrin in rats. Neuroscience.

[11] Chambers-Richards T., Su Y., Chireh B., D’Arcy C. (2021). Exposure to toxic occupations and their association with Parkinson’s disease: A systematic review with meta-analysis. Rev. Environ. Health.

[12] Graaf L., Boulanger M., Bureau M., Bouvier G., Meryet-Figuiere M., Tual S., Baldi I. (2022). Occupational pesticide exposure, cancer and chronic neurological disorders: A systematic review of epidemiological studies in greenspace workers. Environ. Res..

[13] Mohammadi H., Ghassemi-Barghi N., Malakshah O., Ashari S. (2019). Pyrethroid exposure and neurotoxicity: A mechanistic approach. Arh. Hig. Rada Toksikol..

[14] Nandipati S., Litvan I. (2016). Environmental Exposures and Parkinson’s Disease. Int. J. Environ. Res. Public Health.

[15] Furlong M.A., Paul K.C., Cockburn M., Bronstein J., Keener A., Rosario I.D., Folle A.D., Ritz B. (2020). Ambient Pyrethroid Pesticide Exposures in Adult Life and Depression in Older Residents of California’s Central Valley. Environ. Epidemiol..

[16] Kannarkat G.T., Cook D.A., Lee J.K., Chang J., Chung J., Sandy E., Paul K.C., Ritz B., Bronstein J., Factor S.A. (2015). Common Genetic Variant Association with Altered HLA Expression, Synergy with Pyrethroid Exposure, and Risk for Parkinson’s Disease: An Observational and Case-Control Study. NPJ Parkinsons Dis..

[17] Rice D., Barone S. (2000). Critical periods of vulnerability for the developing nervous system: Evidence from humans and animal models. Environ. Health Perspect..

[18] Dayal M., Parmar D., Ali M., Dhawan A., Dwivedi U.N., Seth P.K. (2001). Induction of rat brain cytochrome P450s (P450s) by deltamethrin: Regional specificity and correlation with neurobehavioral toxicity. Neurotox. Res..

[19] Mani V.M., Asha S., Sadiq A.M.M. (2014). Pyrethroid deltamethrin-induced developmental neurodegenerative cerebral injury and ameliorating effect of dietary glycoside naringin in male wistar rats. Biomed. Aging Pathol..

[20] Agrawal S., Dixit A., Singh A., Tripathi P., Singh D., Patel D.K., Singh M.P. (2015). Cyclosporine A and MnTMPyP alleviate α-synuclein expression and aggregation in cypermethrin-induced Parkinsonism. Mol. Neurobiol..

[21] Vaccari C., El Dib R., Gomaa H., Lopes L.C., de Camargo J.L. (2019). Paraquat and Parkinson’s disease: A systematic review and meta-analysis of observational studies. J. Toxicol. Environ. Health B Crit. Rev..

[22] Van Maele-Fabry G., Hoet P., Vilain F., Lison D. (2012). Occupational exposure to pesticides and Parkinson’s disease: A systematic review and meta-analysis of cohort studies. Environ. Int..

[23] Allen M.T., Levy L.S. (2013). Parkinson’s disease and pesticide exposure—A new assessment. Crit. Rev. Toxicol..

[24] Nasuti C., Fattoretti P., Carloni M., Fedeli D., Ubaldi M., Ciccocioppo R., Gabbianelli R. (2014). Neonatal exposure to permethrin pesticide causes lifelong fear and spatial learning deficits and alters hippocampal morphology of synapses. J. Neurodev. Disord..

[25] Carloni M., Nasuti C., Fedeli D., Montani M., Vadhana M.S.D., Amici A., Gabbianelli R. (2013). Early life permethrin exposure induces long-term brain changes in Nurr1, NF-kB and Nrf-2. Brain Res..

[26] Vadhana M.S.D., Carloni M., Nasuti C., Fedeli D., Gabbianelli R. (2011). Early life permethrin insecticide treatment as origin of heart damage in adult rats. Exp. Gerontol..

[27] Carloni M., Nasuti C., Fedeli D., Montani M., Amici A., Vadhana D.M.S., Gabbianelli R. (2012). The impact of early life permethrin exposure on development of neurodegeneration in adulthood. Exp. Gerontol..

[28] Bordoni L., Nasuti C., Di Stefano A., Marinelli L., Gabbianelli R. (2019). Epigenetic memory of early-life parental perturbation: Dopamine decrease and DNA methylation changes in offspring. Oxid. Med. Cell. Longev..

[29] Bordoni L., Nasuti C., Fedeli D., Galeazzi R., Laudadio E., Massaccesi L., Lopez-Rodas G., Gabbianelli R. (2019). Early impairment of epigenetic pattern in neurodegeneration: Additional mechanisms behind pyrethroid toxicity. Exp. Gerontol..

[30] Rossnerova A., Izzotti A., Pulliero A., Bast A., Rattan S.I.S., Rossner P. (2020). The Molecular Mechanisms of Adaptive Response Related to Environmental Stress. Int. J. Mol. Sci..

[31] Pickering A.M., Staab T.A., Tower J., Sieburth D., Davies K.J.A. (2013). A conserved role for the 20S proteasome and Nrf2 transcription factor in oxidative stress adaptation in mammals, *Caenorhabditis elegans* and *Drosophila melanogaster*. J. Exp. Biol..

[32] Bordoni L., Fedeli D., Nasuti C., Capitani M., Fiorini D., Gabbianelli R. (2017). Permethrin pesticide induces NURR1 up-regulation in dopaminergic cell line: Is the pro-oxidant effect involved in toxicant-neuronal damage?. Comp. Biochem. Physiol. Part C Toxicol. Pharmacol..

[33] Rabhi K.K., Esancy K., Voisin A., Crespin L., Le Corre J., Tricoire-Leignel H., Anton S., Gadenne C. (2014). Unexpected effects of low doses of a neonicotinoid insecticide on behavioral responses to sex pheromone in a pest insect. PLoS ONE.

[34] Pickering A.M., Vojtovich L., Tower J., Davies K.J.A. (2013). Oxidative stress adaptation with acute, chronic, and repeated stress. Free Radic. Biol. Med..

[35] Beghoul A., Kebieche M., Gasmi S., Chouit Z., Amiour C., Lahouel A., Soulimani R. (2017). Impairment of mitochondrial integrity and redox status in brain regions during a low-dose long-term exposition of rats to pyrethrinoïds: The preventive effect of quercetin. Environ. Sci. Pollut. Res..

[36] Arora D., Siddiqui M.H., Sharma P.K., Singh S.P., Tripathi A., Mandal P., Singh U.S., Singh P.K., Shukla Y. (2016). Evaluation and physiological correlation of plasma proteomic fingerprints for deltamethrin-induced hepatotoxicity in Wistar rats. Life Sci..

[37] Barlow S.M., Sullivan F.M., Lines J. (2001). Risk assessment of the use of deltamethrin on bednets for the prevention of malaria. Food Chem. Toxicol..

[38] Singh A., Yadav S., Srivastava V., Kumar R., Singh D., Sethumadhavan R., Parmar D. (2013). Imprinting of cerebral and hepatic cytochrome P450s in rat offsprings exposed prenatally to low doses of cypermethrin. Mol. Neurobiol..

[39] Nasuti C., Gabbianelli R., Falcioni M.L., Di Stefano A., Sozio P., Cantalamessa F. (2007). Dopaminergic system modulation, behavioral changes, and oxidative stress after neonatal administration of pyrethroids. Toxicology.

[40] Paxinos G., Watson C. (1998). The Rat Brain in Stereotaxic Coordinates.

[41] Lowry O.H., Rosebrough N.J., Farr A.L., Randall R.J. (1951). Protein measurement with the Folin phenol reagent. J. Biol. Chem..

[42] Concetti A., Massei P., Rotilio G., Brunori M., Rachmilewitz E.A. (1976). Superoxide dismutase in red blood cells: Method of assay and enzyme content in normal subjects and in patients with β-thalassemia (major and intermedia). J. Lab. Clin. Med..

[43] Misra H.P., Fridovich I. (1972). The generation of superoxide radical during the autoxidation of hemoglobin. J. Biol. Chem..

[44] Luck H., Bergmayer M.V. (1974). Catalase. Method of Enzymatic Analysis.

[45] Butler R.N., Butler W.J., Moraby Z., Fettmann M.J., Khoo K.K., Roberts-Thomson I.C. (1994). Glutathione concentrations and glutathione S-transferase activity in human colonic neoplasms. J. Gastroenterol. Hepatol..

[46] Takahashi M., Shibata M., Niki E. (2001). Estimation of lipid peroxidation of live cells using a fluorescent probe, diphenyl-1-pyrenylphosphine. Free Radic. Biol. Med..

[47] Soglia F., Petracci M., Ertbjerg P. (2016). Novel DNPH-based method for determination of protein carbonylation in muscle and meat. Food Chem..

[48] Pham-Huy L.A., He H., Pham-Huy C. (2008). Free radicals, antioxidants in disease and health. Int. J. Biomed. Sci..

[49] Sies H. (2021). Oxidative eustress: On constant alert for redox homeostasis. Redox Biol..

[50] Miao L., St Clair D.K. (2009). Regulation of superoxide dismutase genes: Implications in disease. Free Rad. Biol Med..

[51] Hermes-Lima M., Storey J.M., Storey K.B., Storey K.B., Storey J.M. (2001). Antioxidant defenses and animal adaptation to oxygen availability during environmental stress. Cell and Molecular Response to Stress.

[52] Pamok S., Aengwanich W., Komutrin T. (2009). Adaptation to oxidative stress and impact of chronic oxidative stress on immunity in heat-stressed broilers. J. Therm. Biol..

[53] Haddad J.J., Harb H.L. (2005). L-gamma-Glutamyl-L-cysteinyl-glycine (glutathione; GSH) and GSH-related enzymes in the regulation of pro- and anti-inflammatory cytokines: A signaling transcriptional scenario for redox(y) immunologic sensor(s)?. Mol. Immunol..

[54] Forman H.J., Zhang H., Rinna A. (2009). Glutathione: Overview of its protective roles, measurement, and biosynthesis. Mol. Aspects Med..

[55] Deneke S.M., Fanburg B.L. (1989). Regulation of cellular glutathione. Am. J. Physiol. Cell. Mol. Physiol..

[56] Halliwell B. (1999). Antioxidant defence mechanisms: From the beginning to the end (of the beginning). Free Radic. Res..

[57] Limón-Pacheco J., Gonsebatt M.E. (2009). The role of antioxidants and antioxidant-related enzymes in protective responses to environmentally induced oxidative stress. Mutat. Res. Toxicol. Environ. Mutagen..

[58] Kehm R., Baldensperger T., Raupbach J., Höhn A. (2021). Protein oxidation—Formation mechanisms, detection and relevance as biomarkers in human diseases. Redox Biol..

[59] Gonos E.S., Kapetanou M., Sereikaite J., Bartosz G., Naparło K., Grzesik M., Sadowska-Bartosz I. (2018). Origin and pathophysiology of protein carbonylation, nitration and chlorination in age-related brain diseases and aging. Aging.

[60] Suzuki Y.J., Carini M., Butterfield D.A. (2010). Protein carbonylation. Antioxid. Redox Signal..

[61] Chondrogianni N., Tzavelas C., Pemberton A.J., Nezis I.P., Rivett A.J., Gonos E.S. (2005). Overexpression of Proteasome β5 Assembled Subunit Increases the Amount of Proteasome and Confers Ameliorated Response to Oxidative Stress and Higher Survival Rates. J. Biol. Chem..

[62] Ciechanover A. (1998). The ubiquitin–proteasome pathway: On protein death and cell life. EMBO J..

[63] De Martino G.N., Slaughter C.A. (1999). The proteasome, a novel protease regulated by multiple mechanisms. J. Biol. Chem..

[64] Burré J., Sharma M., Südhof T.C. (2018). Cell Biology and Pathophysiology of α-Synuclein. Cold Spring Harb Perspect Med..

[65] Henderson M.X., Trojanowski J.Q., Lee V.M. (2019). α-Synuclein pathology in Parkinson’s disease and related α-synucleinopathies. Neurosci Lett..

[66] Guo C., Sun L., Chen X., Zhang D. (2013). Oxidative stress, mitochondrial damage and neurodegenerative diseases. Neural Regen. Res..

[67] Courte J., Bousset L., Von Boxberg Y., Villard C., Melki R., Peyrin J.-M. (2020). The expression level of alpha-synuclein in different neuronal populations is the primary determinant of its prion-like seeding. Sci. Rep..

[68] Sokratian A., Ziaee J., Kelly K., Chang A., Bryant N., Wang S., Xu E., Li J.Y., Wang S.H., Ervin J. (2021). Heterogeneity in α-synuclein fibril activity correlates to disease phenotypes in Lewy body dementia. Acta Neuropathol..

[69] Kou J., Gillette J.S., Bloomquist J.R. (2006). Neurotoxicity in murine striatal dopaminergic pathways following co-application of permethrin, chlorpyrifos, and MPTP. Pestic. Biochem. Physiol..

[70] Mitra S., Chakrabarti N., Bhattacharyya A. (2011). Differential regional expression patterns of α-synuclein, TNF-α, and IL-1β; and variable status of dopaminergic neurotoxicity in mouse brain after Paraquat treatment. J. Neuroinflammation.

[71] Manning-Bog A.B., McCormack A.L., Li J., Uversky V.N., Fink A.L., Di Monte D.A. (2002). The herbicide paraquat causes up-regulation and aggregation of α-synuclein in mice: Paraquat and α-synuclein. J. Biol. Chem..

[72] Hashimoto M., Hsu L.J., Rockenstein E., Takenouchi T., Mallory M., Masliah E. (2002). α-Synuclein protects against oxidative stress via inactivation of the c-Jun N-terminal kinase stress-signaling pathway in neuronal cells. J. Biol. Chem..

[73] Skibinski G., Hwang V., Ando D.M., Daub A., Lee A.K., Ravisankar A., Modan S., Finucane M.M., Shaby B.A., Finkbeiner S. (2017). Nrf2 mitigates LRRK2-and α-synuclein–induced neurodegeneration by modulating proteostasis. Proc. Natl. Acad. Sci. USA.

